# Role of IL-17 in LPS-induced acute lung injury: an *in vivo* study

**DOI:** 10.18632/oncotarget.21474

**Published:** 2017-10-04

**Authors:** Qi Ding, Gao-Qin Liu, Yuan-Yuan Zeng, Jian-Jie Zhu, Ze-Yi Liu, Xueguang Zhang, Jian-An Huang

**Affiliations:** ^1^ Department of Respiratory Medicine, The First Affiliated Hospital of Soochow University, Suzhou 215006, China; ^2^ Clinical Immunology Laboratory of Jiangsu Province, Suzhou 215006, China; ^3^ Affiliated Suzhou Hospital of Nanjing Medical University, Suzhou 215006, China

**Keywords:** IL-17, acute respiratory distress syndrome, acute lung injury, RORγt, PI3K pathway

## Abstract

To assess the clinical significance of IL-17 in patients with sepsis-induced acute respiratory distress syndrome (ARDS) and to investigate the effects of IL-17 blocking in a mouse model of acute lung injury (ALI). Significantly increased IL-17 level was found in patients with sepsis-related ARDS compared to healthy controls, whereas significantly increased plasma IL-17 level was also observed in non-survivors compared to that in survivors. According to the data from the mouse ALI model, we found significantly increased IL-17 level in lung tissue lysates, mouse bronchoalveolar lavage fluid (mBALF) and plasma at 6, 12 and 24 h after ALI. Histological analyses revealed that reduced sign of pathological changes and lung injury score in the lungs at 48 h after IL-17 blocking antibody administration. Reduced level of proinflammatory tumor necrosis factor α and increased level of anti-inflammatory factor interleukin-10 were found in both mBALF and plasma. Moreover, IL-17 blocking antibody administration attenuated the expression of RORγt and activity of PI3K-Akt pathway. Increased IL-17 was presented in patients with sepsis-induced ARDS and IL-17 may serve as a biomarker to indicate the severity of ARDS. Moreover, IL-17 antibody administration could relieve the ALI symptom by affecting RORγt level and PI3K pathway.

## INTRODUCTION

Acute lung injury (ALI) and its severe form acute respiratory distress syndrome (ARDS) are severe inflammatory reactions in the lung, which could cause alveolar damage and result in varied degrees of ventilation-perfusion mismatch, severe hypoxemia, decreased lung compliance and noncardiogenic pulmonary edema [1]. Despite various therapeutic strategies (e.g. lung-protective ventilation strategies [2], prone position [3] and fluid-conservative therapy [4]) have been proposed in the last decade, the mortality of ALI/ARDS remain as high as 40% [5]. Therefore, it is necessary to perform the mechanisms related studies that could be helpful in the therapeutic intervention of ALI.

It is generally accepted that the pathogenesis of ALI was caused by lung inflammation and cell apoptosis, including inflammatory cell accumulation, the aberrant level of proteases, reactive oxygen species (ROS), proinflammatory cytokines, dysfunction of alveolar capillary barrier, and death of pulmonary cells [6–8]. IL-17 is a proinflammatory cytokine produced by the memory CD4 + T cells after activation and has been shown to be invovled in the pulmonary cell emigration under the condition of both local gram-negative bacterial infection [9] and antigenic stimuli [10]. IL-17 overexpression or recombinant IL-17 administration into the lung could result in upregulation of chemokines, which recruit inflammatory cells to the airway [11]. Currently, IL-17 is considered to be an attractive target for inflammatory responses in the lung [12]. However, the exact role of IL-17 and Th17 related responses in ALI have not been defined yet.

Here, we assessed the clinical significance of IL-17 in patients with sepsis-induced ARDS and to investigate the effects and mechanism of IL-17 antibody administration in a mouse model of ALI. Our results showed that increased IL-17 was presented in patients with sepsis-induced ARDS and IL-17 may serve as a biomarker to indicate the severity of ARDS. Moreover, IL-17 blocking antibody administration could relieve the ALI symptom by affecting RAR-related orphan receptor gamma t (RORγt) level and phosphoinositide 3-kinase (PI3K) pathway.

## RESULTS

### Increased plasma IL-17 level was presented in patients with sepsis-related ARDS

**Figure 1:**
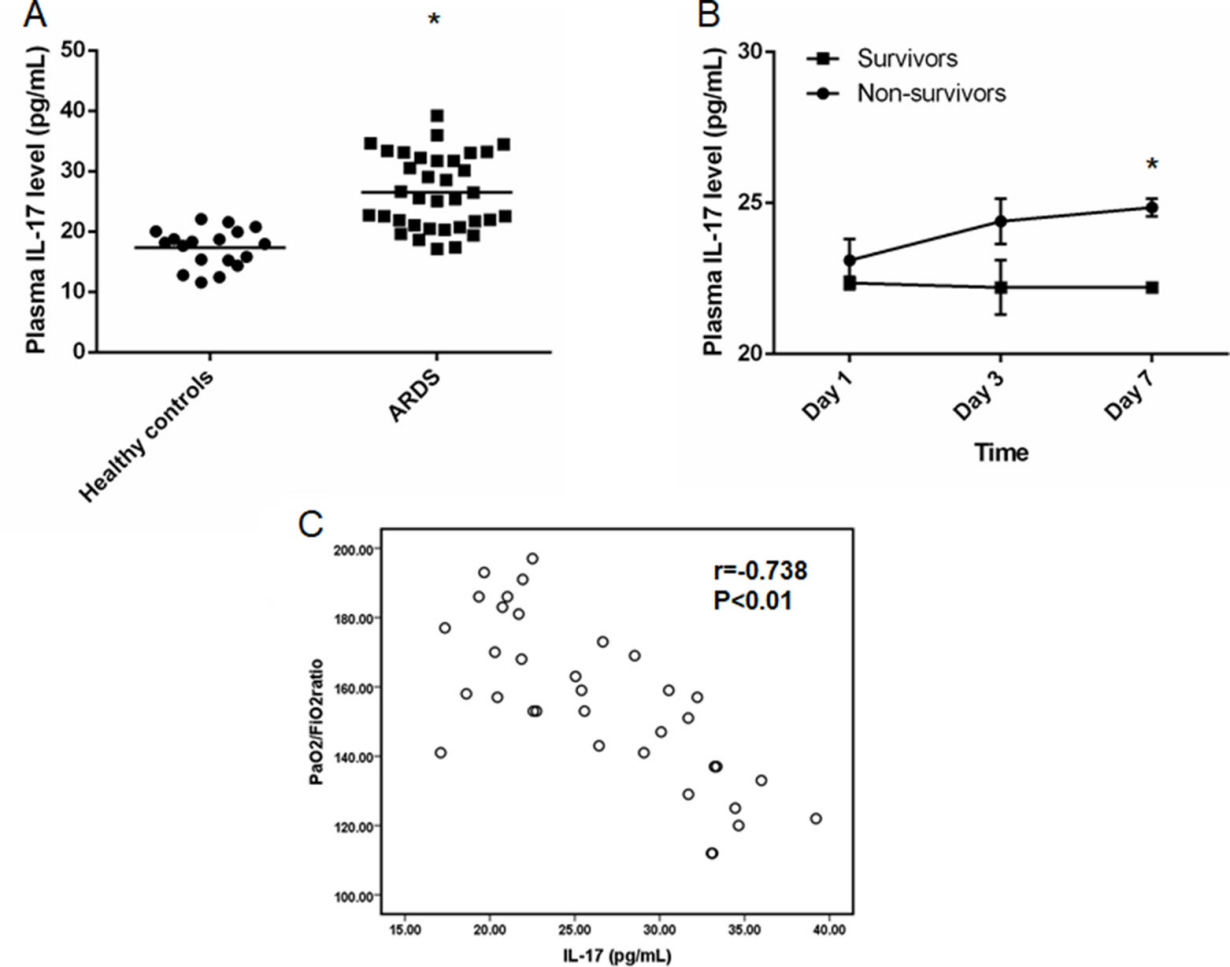
Elevated plasma IL-17 level in patients with sepsis-related ARDS (**A**) Significantly increased IL-17 level in patients with sepsis-related ARDS (*n* = 35) compared to healthy controls (*n* = 18) (*p* < 0.05). (**B**) Plasma IL-17 level for survivors (*n* = 10) and non-survivors (*n* = 8) at different time points. Significantly increased plasma IL-17 level was found in non-survivors compared to that in survivors (*p* < 0.05). (**C**) Negative correlation was found between IL-17 level and PaO_2_/FiO_2_ ratio (r = -0.738, *p* < 0.01). *represents *p* < 0.05 after comparison between 2 groups.

A total of 35 sepsis-related-ARDS patients were included and the plasma level of IL-17 were determined by ELISA. According to the results, we found that significantly increased plasma IL-17 in these patients compared to healthy controls (ARDS: [26.5 ± 6.02] pg/mL vs. Healthy controls: [17.3 ± 3.07] pg/mL, *p* < 0.05) (Figure 1A). Furthermore, we also determined the plasma IL-17 level in sur*vivo*rs and non-sur*vivo*rs at the different time points and significantly increased IL-17 was found in non-sur*vivo*rs when compared to sur*vivo*rs at Day 7 ([24.9 ± 0.30] pg/mL vs. [22.2 ± 0.15] pg/mL, *p* < 0.05) (Figure 1B). In addition, we also found a correlation between plasma IL-17 level and the PaO_2_/FiO_2_ ratio of ARDS patients (r = −0.738, *p* < 0.01) (Figure 1C). The baseline characteristics and clinical data are shown in the supplement data (Supplementary Tables 1 and 2).

### Increased IL-17 level was found in a mouse model of ALI

**Figure 2:**
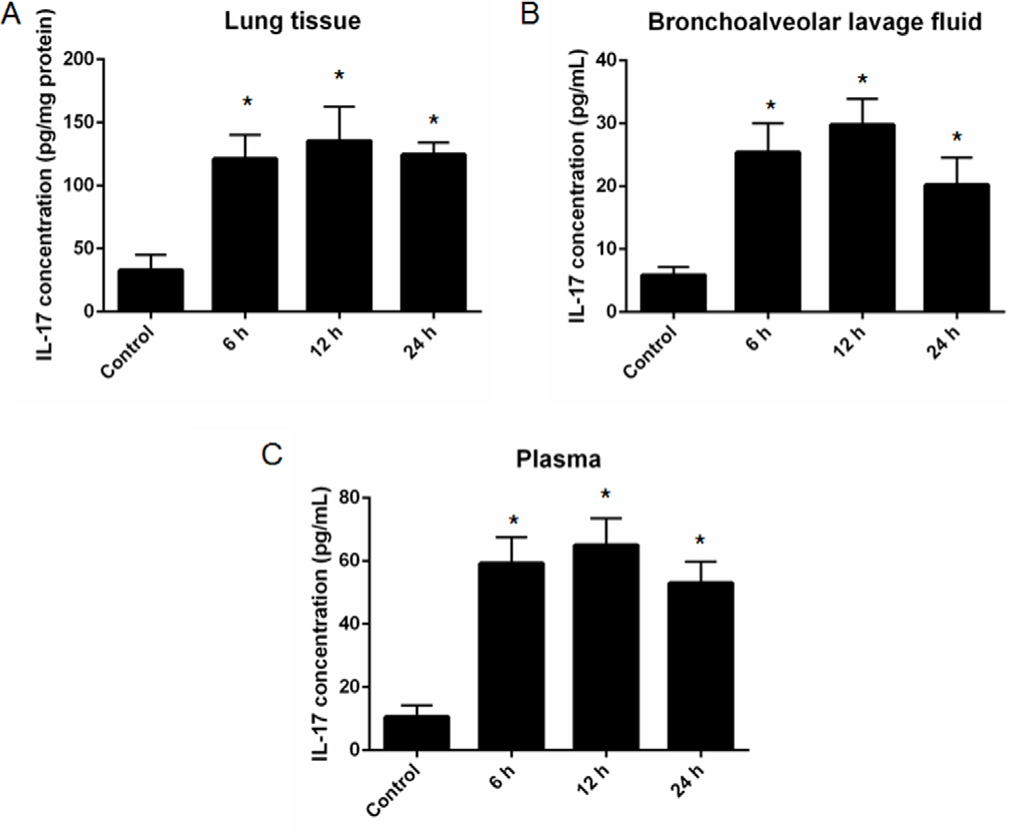
Measurement of IL-17 level in different samples collected from a mouse model of acute lung injury (ALI) Significantly increased IL-17 level was found in lung tissue lysates (**A**, *n* = 3), mouse bronchoalveolar lavage fluid (**B**, *n* = 3) and plasma (**C**, *n* = 3) at 6, 12 and 24 h after ALI . *represents *p* < 0.05 compared to the control mice which were challenged with physiological saline.

We then established a mouse of ALI and determined IL-17 level from different source of sample, including lung tissue, mBALF and plasma. The results showed that significantly increased IL-17 level was found in lung tissue lyse (Control: 6 h: 12 h: 24 h = (32.96 ± 12.26) : (121.3 ± 18.71) :(135.5 ± 27.1) : (124.5 ± 9.68) pg/mg protein; all the *p* < 0.05 vs. control) (Figure 2A), mBALF (Control: 6 h: 12 h: 24 h = (5.9 ± 1.32): (25.4 ± 4.67): (29.8 ± 4.07): (20.2 ± 4.31) pg/mL; all the *p* < 0.05 vs. control) (Figure 2B) and plasma (Control: 6h: 12h: 24 h = (10.6 ± 3.61) : (59.3 ± 8.19): (65.1 ± 8.43): (53.0 ± 6.75) pg/mL; all the *p* < 0.05 vs. control) (Figure 2C) at 6, 12 and 24 h after ALI when compared to the non-ALI controls.

### Effects of IL-17 blocking on LPS-induced ALI *in vivo*

**Figure 3:**
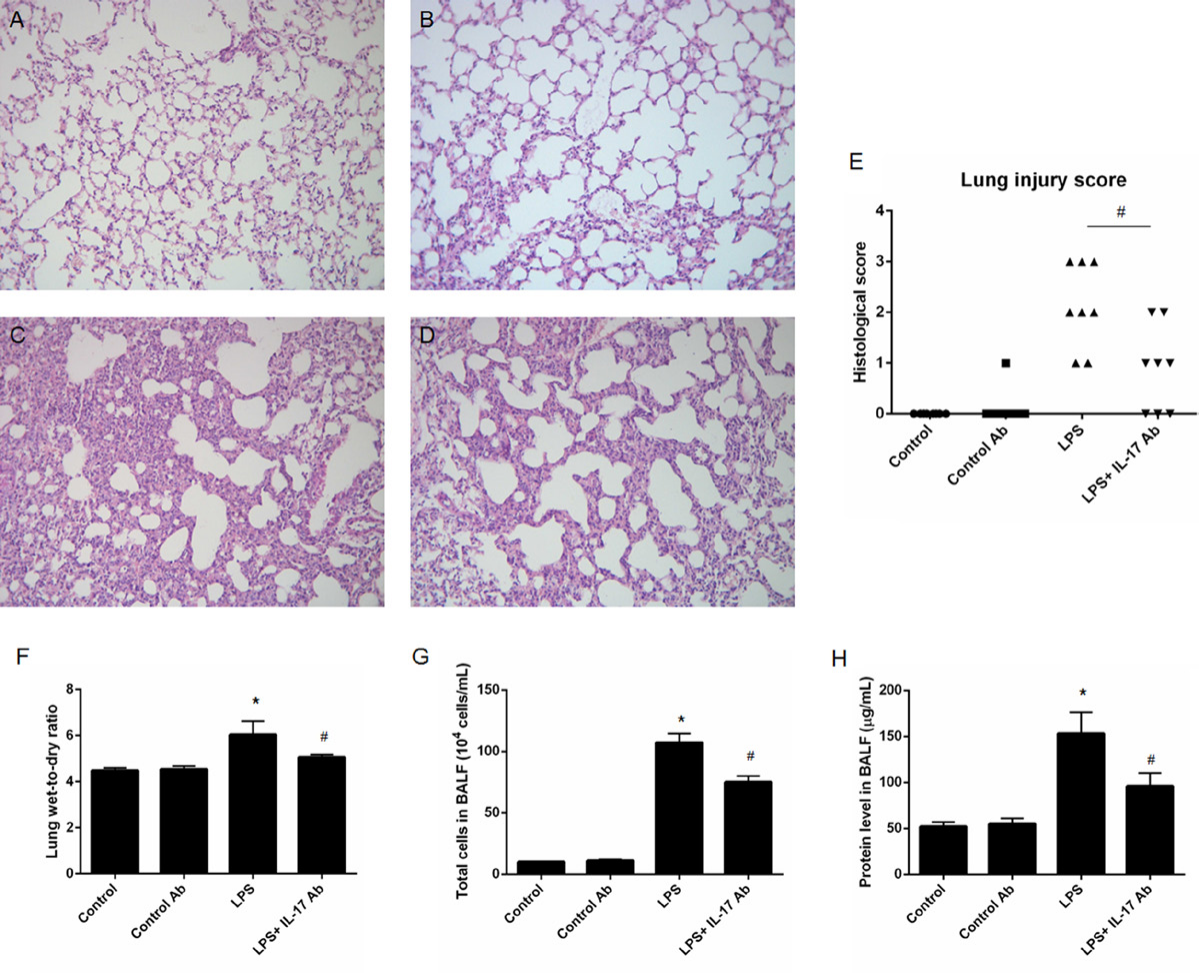
Effects of IL-17 blocking antibody administration on LPS-induced acute lung injury (ALI) *in vivo* Histological analysis of lung tissue collected from the ALI mice at 24 post-injury and control mice. (**A**) Control mice; (**B**) Mice treated with control antibody; (**C**) LPS-induced ALI mice; (**D**) LPS-induced ALI mice treated with IL-17 mAb. (**E**) Lung injury score; (**F**) Lung wet-to-dry ratio; (**G**) Total cell in mouse bronchoalveolar lavage fluid (mBALF) collected from different groups of mice; (**H**) Protein level in mBALF collected from different groups of mice. All the pathological images were 40× magnification. * represents *p* < 0.05 when compared to the mice treated with control antibody while # represents *p* < 0.05 when compared to LPS-induced ALI mice.

Histological analysis (Figure 3A–3D) and lung injury score assessment (Figure 3E) of lung tissues showed that IL-17 antibody administration could significantly decrease injury such as pulmonary edema, hemorrhage and leukocytes infiltration into alveoli compared with mice in control group. IL-17 antibody administration could significantly decrease lung wet-to-dry ratio (LPS vs. LPS + IL-17 Ab = 6.04 ± 0.58 vs. 5.06 ± 0.11, *p* < 0.05 ) (Figure 3F), total cells (LPS vs. LPS + IL-17 Ab = [104 ± 7.2] × 10^4^ vs. [75 ± 5.0] ×10^4^ cells/mL , *p* < 0.05 ) (Figure 3G) and protein level (LPS vs. LPS + IL-17 Ab = [153 ± 23] vs. [96 ± 14] µg/mL, *p* < 0.05 ) (Figure 3H) in mBALF in mice from LPS + IL-17 Ab group compared to mice in LPS group.

### IL-17 blocking decrease the level of inflammatory cytokines in ALI

**Figure 4:**
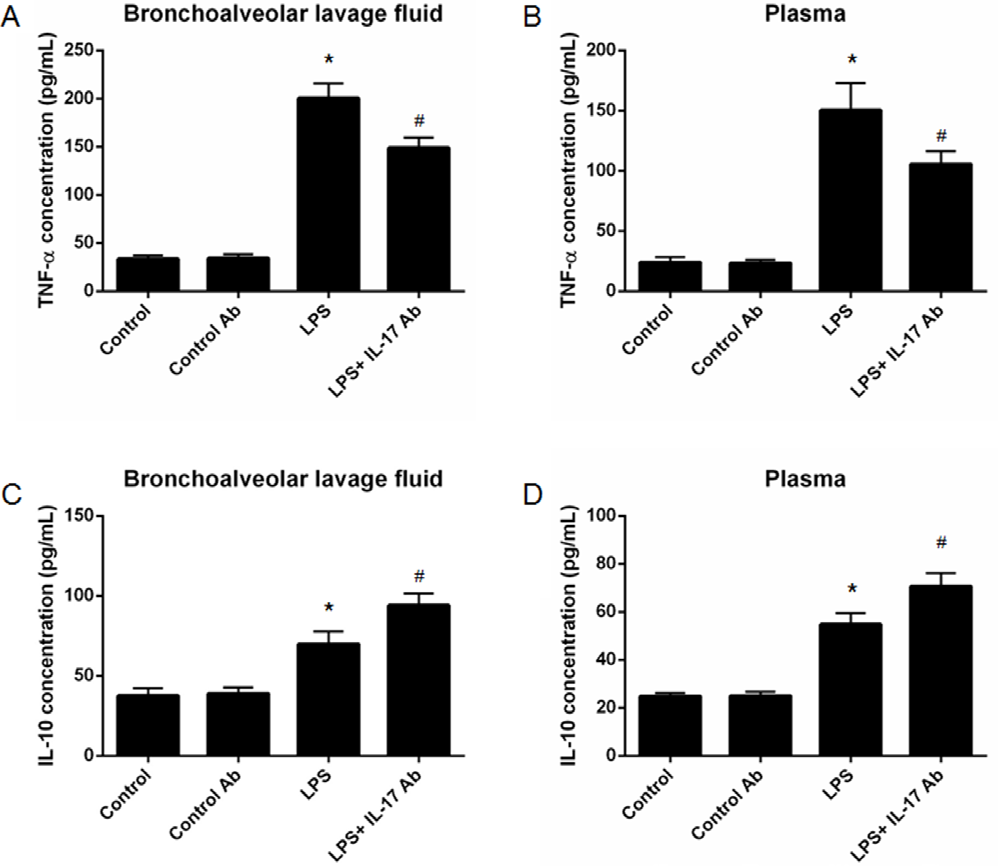
Measurement of inflammatory cytokines in mouse bronchoalveolar lavage fluid (mBALF) and plasma IL-17 blocking antibody administration could significantly decrease the tumor necrosis factor-α (TNF-α) level in mBALF (**A**) and plasma (**B**). IL-17 blocking antibody administration could significantly increase interleukin-10 (IL-10) level in mBALF (**C**) and plasma (**D**). *represents *p* < 0.05 when compared to the mice treated with control antibody while # represents *p* < 0.05 when compared to LPS-induced ALI mice.

We then measured the inflammatory cytokines in mBALF and plasma in different group of mice. The results showed that IL-17 antibody administration could significantly decrease the TNF-α level in mBALF (LPS vs. LPS + IL-17 Ab = [200.5 ± 15.50] vs. [148.8 ± 10.63] pg/mL, *p* < 0.05) (Figure 4A) and plasma (LPS vs. LPS + IL-17 Ab = [150.4 ± 22.67] vs. [105.6 ± 10.61] pg/mL, *p* < 0.05) (Figure 4B) in mice from LPS + IL-17 Ab group compared to mice in LPS group. Moreover, IL-17 blocking antibody administration could also significantly increase IL-10 level in mBALF (LPS vs. LPS + IL-17 Ab = [70.0 ± 7.80] vs. [94.1 ± 7.38] pg/mL, *p* < 0.05) (Figure 4C) and plasma (LPS vs. LPS + IL-17 Ab = [54.9 ± 4.63] vs. [70.6 ± 5.61] pg/mL, *p* < 0.05) (Figure 4D) in mice from LPS + IL-17 Ab group compared to mice in LPS group.

### RORγt and activity of PI3K-Akt pathway were involved in the effects of IL-17 antibody administration

**Figure 5:**
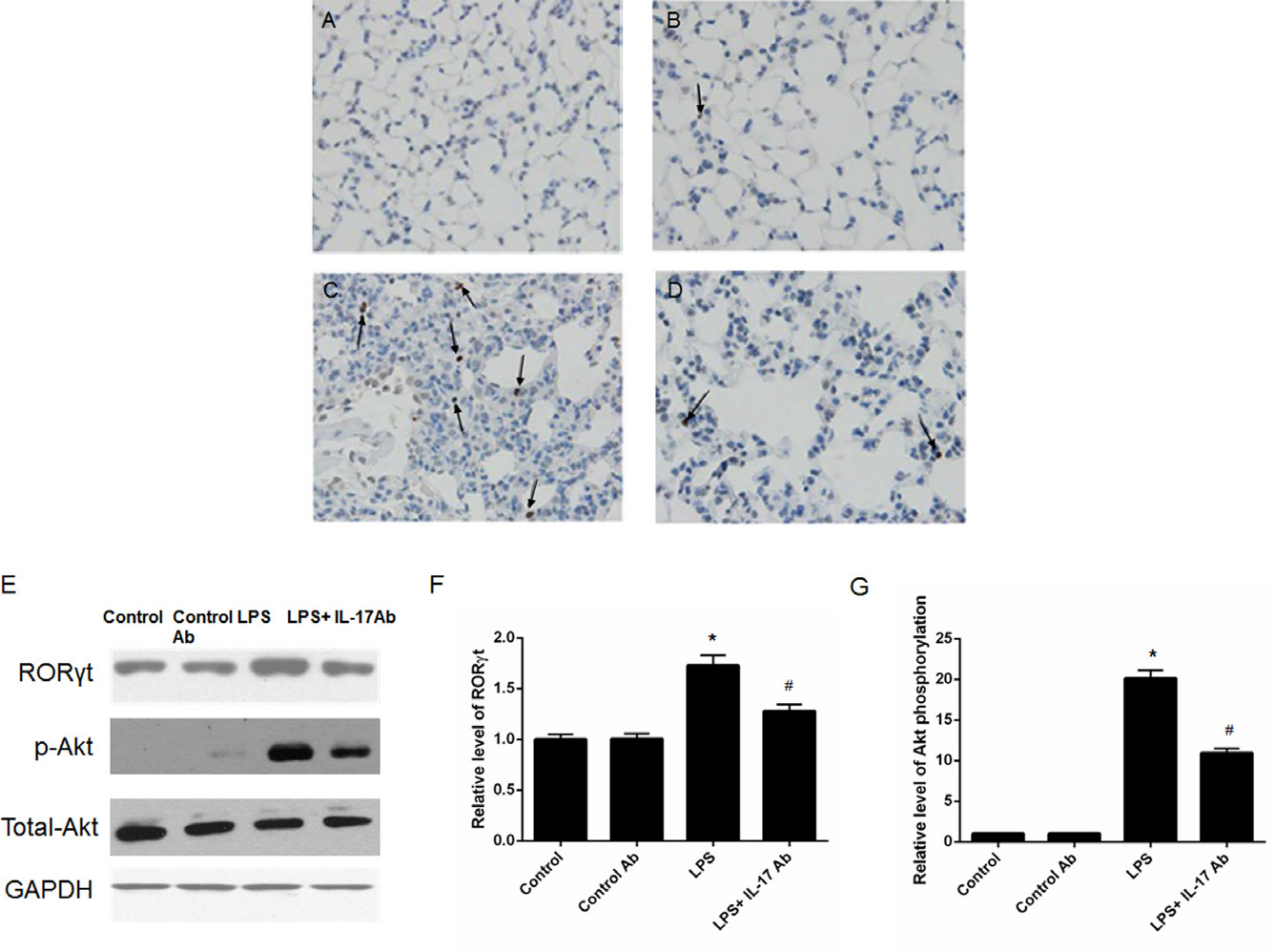
IL-17 blocking attenuated the expression of RORγt and activity of PI3K-Akt pathway Immunohistological analysis of Th-17 related key transcription factor RORγt. (**A**) Control mice; (**B**) Mice treated with control antibody; (**C**) LPS-induced ALI mice; (**D**) LPS-induced ALI mice treated with IL-17 blocking antibody. (**E**) Western-blot analysis of RORγt and activity of PI3K-Akt pathway. IL-17 blocking antibody administration could decrease the expression of RORγt and Akt phosphorylation. (**F**) Semi-quantitation of the RORγt level. (**G**) Semi-quantitation of the Akt phoshorylation level. The arrows showed the positive staining of RORγt.* represents *p* < 0.05 when compared to the mice treated with control antibody while # represents *p* < 0.05 when compared to LPS-induced ALI mice.

To further explore the mechanism, we performed immunohistological analysis of Th-17 related key transcription factor RORγt and employed Western-blot to analyze the RORγt and activity of PI3K-Akt pathway. The results showed that IL-17 blocking antibody administration could decrease the expression of RORγt (Figure 5A–5D by immunohistological assay and *p* < 0.05 according to Figure 5E and 5F by western-blotting) and Akt phosphorylation (*p* < 0.05; Figure 5E and 5G by western blotting) were found in in mice from LPS + IL-17 Ab group compared to mice in LPS group.

## DISCUSSION

As a form of inflammatory disease, ALI could cause alveolar damage and result in varied degrees of ventilation-perfusion mismatch, severe hypoxemia, decreased lung compliance and noncardiogenic pulmonary edema, and finally even death. Recent studies have suggested that the immune regulation disorder may be an important factor in initiating inflammation. Treg and Th17 cells, which belong to CD4 + T cells, have gained much attention. Treg, characterized as a CD4 + CD25 + FOXP3 + T cell, is the master of immune system through a) suppressing a wide array of effector immune cells, including Th cells, B cells dendritic cells and macrophages, and b) secreting of immunosuppressive cytokines, such as TGF-β and IL-10 [13, 14]. Th17 cells, a recently detected effective subset of CD4 + T cells and the major source of IL-17, play an important role in defending a host against microorganisms, such as staphylococcus [15, 16]. A previous study showed that patients with infection-induced ALI/ARDS appeared to have obvious activation and proliferation of T-cells, particularly the presence of Th17 cells [17]. However, the exact role of Th17 has not been determined yet.

In present study, our results demonstrated that significantly increased IL-17 level in patients with sepsis-related ARDS compared to healthy controls while significantly increased plasma IL-17 level was found in non-sur*vivo*rs compared to that in sur*vivo*rs (In the sur*vivo*r and non-sur*vivo*rs setting, we only detected 18 patients due to the first 17 patients was lost to follow when we performed the Day 3 and Day 7 plasma collection). Significantly increased IL-17 level was found in lung tissue lysate, mBALF and plasma at 6, 12 and 24 h after ALI. Histological analyses revealed that reduced sign of pathological changes in the lungs after IL-17 blocking antibody administration. Reduced level of proinflammatory TNF-α and increased level of anti-inflammatory factor IL-10 were found in both mBALF and plasma. Moreover, IL-17 blocking antibody administration attenuated the expression of RORγt and activity of PI3K-Akt pathway. To the best of our knowledge, this is the first *in vivo* study to elucidate the role of IL-17 in ALI.

In human ARDS patients, very little has been reported related to IL-17. Recently, Li et al showed that found significant elevation of IL-17A in BALF from patients with ARDS and recombinant IL-17A directly increased permeability across cultured human alveolar epithelial monolayers [18]. Furthermore, Yan et al. also detected increased plasma IL-17 level in ARDS patients [19]. Here, we obtained the similar results as these study, we found increased IL-17 level in ARDS patients and correlation between IL-17 level and ARDS survival. In addition, we detected a significantly increased IL-17 level in mBALF from ALI mice.

The differentiation of Th17 cells is moderated by many pathways. RORγt is one of the most important nuclear transcription factors in this regulation process [20, 21]. Here, we examined the expression of RORγt in lung tissue by immunohistological analysis and western-blot. Both results suggested that ALI could increase the expression of RORγt. Moreover, our results also indicated that IL-17 mAb treatment could attenuate the severity of ALI by affecting RORγt. In addition, Studies have shown that PI3K-Akt signaling positively regulates Th17 differentiation via multiple mechanisms including the regulation of hypoxia-inducible factor 1α (HIF-1 α) expression, Signal transducer and activator of transcription 3 (STAT3) phosphorylation, Gfi1 downregulation, and the nuclear translocation of RORγt [22–24]. Therefore, the Akt phosphorylation could be considered as the initiator during the Th17 differentiation. Here, our results showed that decreased Akt phosphorylation in IL-17 mAb treated ALI mice, supporting the involvement of PI3K-Akt signaling during Th17 cell differentiation.

## MATERIALS AND METHODS

### Ethical approval of the study protocol

All research involving human participants was approved by the Institutional Review Board of the First Affiliated Hospital of Soochow University School of Medicine, Suzhou, China and written informed consent was obtained from each participating individual. The study protocol was also approved by the Institutional Animal Care and Use Committee of Soochow University School of Medicine as adherent to generally accepted international guidelines for animal experimentation.

### Patients

The cohort consisted of 35 sepsis-related-ARDS patients admitted to the respiratory intensive care unit (RICU) between June 1, 2012 and May 31, 2015 at the First Affiliated Hospital of Soochow University. The inclusion criteria, primarily as previously described [25], were performed according to the Berlin definition [26]. The exclusion criteria were as follows: 1) 18 years of age and younger, 2) previously underwent immunosuppressant therapy, 3) received long term glucocorticoid therapy (0.5 mg/Kg/D, ≥ 1 months), 4) confirmed or suspected malignant tumor history, and 5) previously included in other studies. Blood samples were collected within 24 hours (*n* = 35) and at another 2 time points (*n* = 18) after the patient met the inclusion criteria. Briefly, peripheral venous blood samples were drawn on an agreed upon time point and centrifuged at 3000 rpm for 15 minutes. The plasma was stored at −80°C for ELISA testing. The plasma levels of IL-17 (Shanghai Yanhui Biological Technology Co., Ltd) was measured using commercially available sandwich ELISA kits.

### Animals

Male C57BL/6 mice (6–8 weeks old) were purchased from the Experimental Animal Center of the Soochow University and were maintained in cages at temperature of 24–26°C under a 12 hr light/dark cycle with free access to food and water.

### Acute lung injury (ALI) model and mice grouping

Mice were randomly divided into 4 groups as follows: Control group (PBS, *n* = 8), Control antibody group (treated with 1µg control antibody, *n* = 8), LPS group (treated with 3 mg/kg LPS, *n* = 8) and LPS + IL-17 antibody (treated with 3 mg/kg LPS and 1µg IL-17 antibody, *n* = 8). After grouping, these mice were anesthetized through intraperitoneal administration of sodium pentbarbital (40 mg/kg) and intratracheally instilled with 3 mg/kg LPS (Sigma, prepared from Escherichia coli 0111:B4) in 50 μ L PBS or sterile PBS alone (as the control group), and control antibody and IL-17 antibody were infused through tail vein injection after LPS instillation for 6 h. The mice in these 4 groups were sacrificed by cervical dislocation at the time point of 24 h post-exposure to evaluate the severity of the ALI. Since all the mice were sacrificed at 24 h post the LPS installation. No mice mortalities was found.

### Mouse bronchoalveolar lavage (mBAL)

mBAL was performed with the whole lung. Three hundred microliter aliquots of 37°C, sterile, pyrogen-free, 0.9% saline were flushed through the tracheotomy tube and this process was repeated for 5 times. The five fractions were recovered and pooled. The total number of cells was counted using a standard hemocytometer. The mBAL fluid (mBALF) was then centrifuged at 600× g for 5 min. The supernatant was collected and stored at −80°C. Protein quantification was determined by using BCA Protein Assay Kit (Pierce, Rockford, IL, USA).

### Lung wet-to-dry weight ratio

To measure the total amount of lung water, the animals were dissected under deep sevoflurane anesthesia, and the lung weight was measured immediately after its excision (wet weight). The lung tissue was then dried in an oven at 60°C for 5 days and re-weighed as dry weight. The wet-to-dry lung weight ratio (W: D ratio) was calculated by dividing the wet weight by the dry weight.

### Histology

Lung from different treatment group were collected 6, 24, 48 h after LPS induced lung injury and fixed with 10% formalin. After fixation, the lungs were embedded in paraffin, cut into 5 μm section and stained with hematoxylin-eosin staining (H&E staining) and observed under by microscopy (Zeiss, Gottingen, Germany). Slides were blinded and scored by an experienced pathologist in a semiquantitative manner according to the relative degree of inflammatory infiltration. Inflammation was scored as follows: 0, no inflammation; 1, perivascular cuff of inflammatory cells; 2, mild inflammation, extending throughout 25% of the lung; 3, moderate inflammation covering 25–50% of the lung; 4, severe inflammation involving over one-half of the lung.

### Immunohistochemistry

Lung tissue were processed and immunostained as previously described [27]. Negative controls, prepared by omission of primary antibody or substitution with a non-immune, isotype control primary antibody were examined to confirm the specificity of primary antibodies. Th17 cells were detected with rabbit anti-RORγt IgG (Cell Signaling Technology, Cambridge, MA, USA) and HRP-conjugated goat anti-rabbit IgG. Finally, the sections were stained with oxidase diaminobenzidine (DAB) (ZSGB-BIO, China) for 5 min, followed by hematoxylin staining for 2 min.

### Measurement of IL-10 and TNF-α

The concentrations of interleukin 10 (IL-10) and Tumor necrosis factor-α (TNF-α) in mBALF and plasma were quantified by enzyme-linked immunosorbent assay (ELISA), using standard commercially available ELISA kits (R&D Systems, Minneapolis, MN, USA).

### Western blotting

Frozen lung tissues obtained from model mice were lysed in RIPA buffer, followed by high-speed centrifugation and quantification using bicinchoninic acid. Cellular proteins were separated by sodium dodecyl sulfate-polyacrylamide gel electrophoresis and transferred onto polyvinylidenedifluoride membranes. After blocking, membranes were incubated with total or phospho-Akt and RORγt monoclonal primary antibodies (Cell Signaling Technology, Cambridge, MA, USA). GAPDH (Santa Cruz Biotechnology, Santa Cruz, CA, USA) was used as the loading control. Appropriate horseradish peroxidase-conjugated secondary antibodies were applied. The protein bands were detected with SuperSignal Ultra Chemiluminescent Substrate (Pierce, Rockford, IL, USA) on X-ray films (Kodak, Tokyo, Japan). The quantitation of western-blot was fulfilled by using Image J software (National Institutes of Health).

### Statistical analysis

All statistical analyses were performed using the SPSS13.0 software (SPSS Inc., Chicago, IL, USA). The results were presented as means ± standard deviation (SD). One way ANOVA or student t test was used to examine the differences among multiple groups or between 2 groups. Correlation analysis was performed by spearman test. *P* < 0.05 was considered as statistically significance.

## CONCLUSIONS

In conclusion, we found here that increased IL-17 was presented in patients with sepsis-induced ARDS and IL-17 may serve as a biomarker to indicate the severity of ARDS. Moreover, IL-17 blocking antibody administration could relieve the ALI symptom by affecting RORγt level and PI3K pathway.

## SUPPLEMENTARY MATERIALS TABLES


