# Supplementary material for: Role of IL-17 in LPS-induced acute lung injury: an in vivo study
Source: Oncotarget. 2017 Oct 4;8(55):93704–11. doi: 10.18632/oncotarget.21474 (PMC5706829; doi:10.18632/oncotarget.21474)
Supplement: Supplementary file 1 [file oncotarget-08-93704-s001.pdf]

Role of IL-17 in LPS-induced acute lung injury: an *in vivo* study

SUPPLEMENTARY MATERIALS

Supplementary Table 1: Baseline characteristics of 35 patients with sepsis-related ARDS and healthy controls

Characteristics	ARDS (*n* =35)	Healthy controls (*n* = 18)	*p*-value
Age, year	65 (53–74)	63 (54–70)	0.60
Male sex, *n* (%)	26 (74.2)	12 (66.6%)	0.55
APACHE II score	21 (17–26)	-	
SAPS II score	43 (33–51)	-	
PaO2/FiO2ratio	162 (120–193)	-	
Hospital stay, day	16 (9–26)	-	
ICU stay, day	8 (3–15)	-	
WBC count, $\times 10^3/mm^3$	13.28 (8.16–19.48)	-	
PCT (pg/ml)	2.53 (0.48–6.57)	-	
Predisposing conditions, *n* (%)			
Bacteremia	12 (34.3)	-	
Pneumonia	23 (65.7)	-	
MODS, *n* (%)			
Dysfunction ≥ 3 organs	10 (28.5)	-	
Septic shock	16 (45.7)	-	
Renal failure	5 (14.3)	-	
Hepatic failure	4 (11.4)	-	

Data are presented as median (IQR) or No. (%).

ARDS = acute respiratory distress syndrome; APACHE = acute physiology and chronic health evaluation; SAPS II= new simplified acute physiology score; MODS = multiple-organ dysfunction syndrome; WBC = white blood cell; PCT= procalcitonin.

Supplementary Table 2: Characteristics of 18 patients followed to D7

Number	Outcome	Gender	Age	Microorganisms	Septic shock
1	S	F	21	Mycobacterium tuberculosis	N
2	S	F	61	Acinetobacter baumannii	N
3	S	F	56	Pseudomonas aeruginosa	N
4	S	M	66	Fungi	N
5	S	M	67	Pseudomonas aeruginosa	N
6	S	M	71	Stenotrophomonas maltophilia	N
7	S	F	64	Acinetobacter baumannii	Y
8	S	M	78	Acinetobacter baumannii	N
9	S	F	70	Bacillus levans	N
10	S	M	55	Pseudomonas aeruginosa	Y
11	NS	M	79	Acinetobacter baumannii	N
12	NS	M	73	Acinetobacter baumannii	Y
13	NS	M	78	Acinetobacter baumannii	Y
14	NS	F	71	Pseudomonas aeruginosa	N
15	NS	M	34	Staphylococcus aureus	Y
16	NS	M	72	Acinetobacter baumannii	N
17	NS	M	42	Virus	Y
18	NS	F	53	Mycobacterium tuberculosis	Y

S = survivors; NS = nonsurvivors; F = female; M = male; Y = yes; N = no.
